# The Cervical Abrasion Index of Treatment Needs (CAITN) Procedure for Population Groups and Individuals

**DOI:** 10.7759/cureus.36324

**Published:** 2023-03-18

**Authors:** Abdul Salam T. A., Sheeja S Varghese, Rekha P Shenoy

**Affiliations:** 1 Department of Public Health Dentistry, Saveetha Dental College and Hospitals, Saveetha Institute of Medical and Technical Sciences, Saveetha University, Chennai, IND; 2 Department of Periodontology, Saveetha Dental College and Hospitals, Saveetha Institute of Medical and Technical Sciences, Saveetha University, Chennai, IND; 3 Department of Public Health Dentistry, Yenepoya Dental College and Hospital, Yenepoya University, Mangalore, IND

**Keywords:** dental indices, cervical abrasion, treatment needs, caitn probe, buccolingual measurements

## Abstract

Because of their complex epidemiology and etiology, cervical abrasions usually manifest with concealing symptoms. The buccolingual dimension of the sore is considered the most important metric to rank the damage and evaluate its long-term prognosis. In this piece, we will break this down and offer the Cervical Abrasion Index of Treatment Needs (CAITN), a simple grouping structure based on the clinical presentation of the sore that may be used to establish a basic, useful, treatment-based order. CAITN is the practical approach to routine screening and recording cervical abrasion lesions. The index provides epidemiologists, public health professionals, and practitioners with a practical means of assessing the treatment needs (TN) of cervical abrasion.

## Introduction and background

The word *abrasion* comes from the Latin word *abrasum*, which describes a pathological process unrelated to caries that occurs when abrasive substances are applied to the tooth surface, causing a loss of tooth substance around the area where the tooth meets the gums and the root [1]. Morphological and histological features of the cervical region contribute to the region's disproportionately high rate of lesion development, where the tooth crown becomes more vulnerable to physical and chemical stimuli as the enamel thickness gradually decreases near the cementoenamel junction (CEJ) and the dentinoenamel junction. In its initial phases, the cervical abrasion appears clinically as a narrow horizontal groove on the buccal/labial surface of the tooth near the CEJ. It also has a polished surface with a glossy appearance, as well as tactile sensitivity to the path of the explorer [2,3].

Much while etiological factors matter when determining a treatment plan, they matter even more at the preventive stage. The treatment method and restoration material increasingly depend on the lesion's actual appearance when the patient arrives at the clinic. Studies have shown that measuring the lesion's buccolingual dimensions is the most effective way to both detect the condition and monitor its progression over time [3]. A literature search reveals that despite the prevalence of abrasions, there is no agreed-upon scientific classification that can be utilized as a discussion point in medical settings [4-6].

Therefore, a categorization of the lesion's therapy based on its clinical presentation is necessary. Restorative material, the operator's clinical expertise, and cavity size all have a role in how long and how well a restoration functions in the mouth. The buccolingual size of the cavity, quantity of tooth structure to be repaired, cosmetic concerns, thickness of the remaining dentin, and the cavity's closeness to the pulp all have a role in determining the restorative material [3]. The authors suggest a strategy based on treatment needs (TN) that begins with a clinical evaluation of the severity of cervical lesions based on buccolingual dimensions.

## Review

Scope and purpose

The quantitative measures of oral diseases most commonly relied on indices. The desirable characteristics of an index are its capacity to be understood and used by all parties involved. Generally speaking, there are three main applications for indices and reporting the prevalence and incidence of a specific ailment [7].

Epidemiology studies of cervical abrasion should use CAITN. It's useful for figuring out what kind of treatment a patient will need and keeping tabs on how well that care is working. The Cervical Abrasion Index of Treatment Needs (CAITN) is a process that takes into account clinical characteristics and criteria while determining the best course of treatment. It can neither be intended for an exhaustive evaluation of the abrasion nor be utilized as a diagnostic tool for planning specific clinical therapy in individual circumstances. It is mostly used as a screening tool for determining the necessity for treatment of cervical abrasions in the community depending on the depth of the lesion. Appropriate dental care services for populations and individuals can be arranged using this data. Socio-economic, personal, and other factors will influence decisions on the priority use of the resources and thus the actual therapeutic care provided. Identification of the magnitude of the need provides an essential basis for promoting primary healthcare programs.

Clinical criteria for evaluating the buccolingual dimension

The pulp is vulnerable if the residual dentin thickness is less than the depth of a carious or noncarious lesion. The extent of a cervical lesion should be confirmed clinically before restoration. The depth of a lesion in the cervical region at the CEJ may range from superficial to moderate to near pulp level, depending on the cause of the damage.

Etiological variables

**Figure 1:**
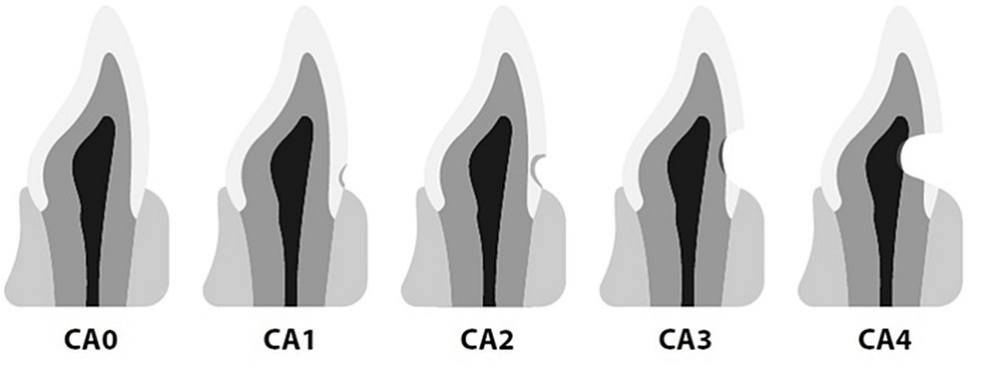
Diagrammatic representation of the depth of the cervical lesions and the corresponding CAITN scores. CAITN, Cervical Abrasion Index of Treatment Needs

Many variables, including rough toothbrushing and the use of dentifrice with a high-abrasive component, may lead to tooth abrasion. Brushing causes lesions that are more noticeable in the incisor, canine, and premolar regions than they are in the molar region [3,8,9]. Figure 1 illustrates the diagrammatic representation of the depth of the cervical lesions and the corresponding CAITN scores.

The symptoms of sensitivity are dependent on the amount of dentin exposed to the environment, the reparative or sclerotic dentin formed, and the proximity to the pulp. However, variations in the buccolingual dimensions cannot establish the severity of cervical abrasions.

Treatment needs

Desensitizing agents, fluoride varnish, and topical casein phosphopeptide amorphous calcium phosphate pastes are routinely used to alleviate pain and boost caries' resistance. If the symptoms of sensitivity are severe, then fluoride varnish applications, desensitizing agents, or electrophoresis should be advocated. A glass ionomer cement (GIC) or composite resin is then used to restore the affected area.

Moderate-to-severe cervical lesions may cause symptoms such as sensitivity to sweets, colds, or air. Sclerotic dentin might cause the lesion to have no noticeable symptoms. Sometimes, the sclerotic dentin has a dark yellow-brown color. Pathological changes occur in the dentin, resulting in a tissue that is chemically and structurally distinct from the healthy dentin. Resin-modified GIC or composite resin liners are placed in these lesions before being repaired with a GIC or a composite resin [10,11]. Certain lesions, especially those that go deeper, might cause discomfort or sensitivity that lasts long after the stimulus has been withdrawn. If the lesion is near the pulp, a resin-modified GI or composite resin repair with a calcium hydroxide liner may be the best option, which is recommended [3,12]. A calcium hydroxide liner is advised if the lesion is rather deep and close to the pulp. Root canal treatment (RCT) is advised when the pulp is exposed and symptoms of irreversible pulpitis are present.

By definition, CAITN only assesses curable illnesses, as indicated by the term *treatment needs*. It should not, however, be used as a precise prescription for clinical therapy. The hierarchy of needs can be used to assess the needs for cervical abrasion care and take or recommend suitable activities. It can be used in the community as part of a comprehensive cervical abrasion control and prevention program. It may entail the use of selective or limited primary or secondary preventative measures. CAITN, when used with these realistic goals in mind, provides an overview of the magnitude of treatment care required to improve the oral health of the individual or community.

The goal of attempting to manage the lesion is to encourage appropriate oral health care, which improves the quality of life, well-being, and aesthetic appearance reduces halitosis and reduces the risk of dental caries [13]. The goal of effective dental care is to promote oral and general health [14].

Choice of age groups

In addition, the teeth of older patients have been exposed to the relevant pathologic variables for a longer period compared to those of younger individuals. Cervical abrasions are expected to increase in frequency going forward. Another factor that increases the incidence of cervical lesions in the elderly is the prevalence of gingival recession and bone loss [15]. This results in more root surface and cementum exposure. Our extensive studies [16,17] show that cervical abrasion is more common among those aged 24 to 53 years. Examination of the population within the age group may thus be justified. The eligible patients were included in the order in which they visited the specialty care, thus forming a convenience sample.

Recording of data

In clinical practice, all the teeth of the patients that meet the inclusion criteria should be recorded. In an epidemiological survey, a box chart is recommended for recording CAITN. The oral cavity was divided into three regions in each arch: premolar, canine, and incisor that are defined by tooth numbers, as shown in the chart. Estimates of cervical abrasion lesions are most accurate when these teeth are used. When evaluating the premolars and incisors, only the highest score from each pair is kept. Every area in the index receives a single score.

Instrument used were the CAITN probe and mouth mirror.

Design of the CAITN Probe

The cervical abrasion of the tooth quantitatively using the newly developed instrument, that is, the CAITN probe. The CAITN probe had a total length of 16.3 cm, weight of 60 g, and graduation range of 0 to 20 mm. The probe consists of a beak with a needlepoint, shank, pivot points, shaft, scale, and several finger rings. The finger rings are round with a 3 cm inner diameter, 4 cm outside diameter, 3 cm breadth, and 2 mm depth. The width of the scale is 1 cm, and the length is 5 cm; both sides have marks from 0 to 20.

**Figure 2:**
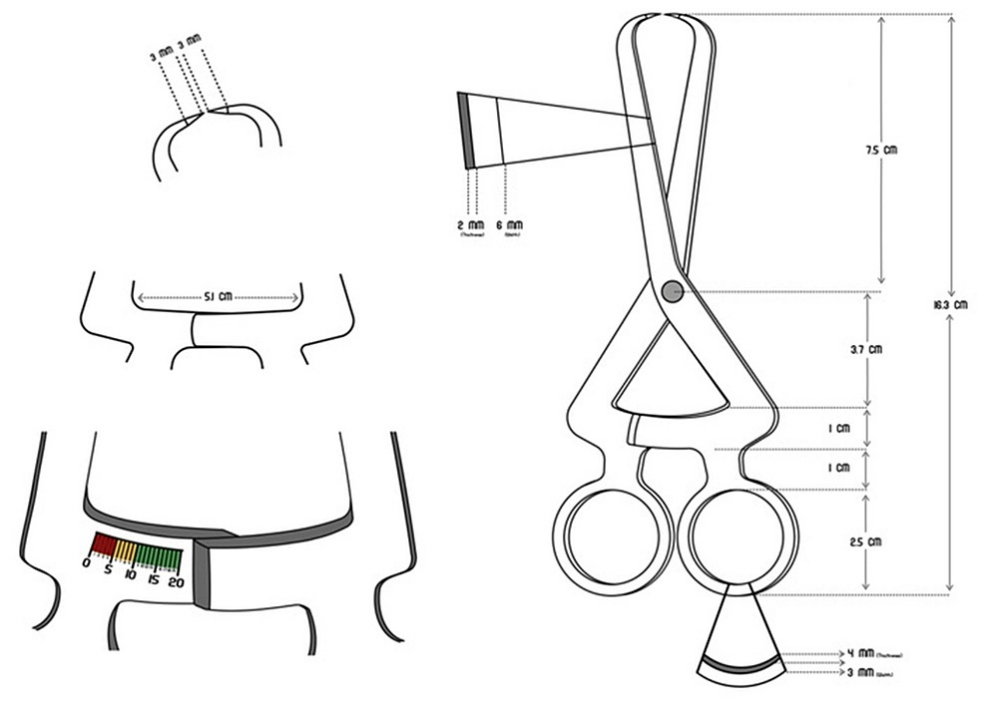
Dimensions of the CAITN probe. Figure credits: Abdul Salam T. A. CAITN, Cervical Abrasion Index of Treatment Needs

The shaft continues the scale and joins with the shank at the pivot points; it is 11 cm long, 3 mm broad, and 1 mm thick. The shank then curves and tapers to create opposing beaks. The 3 mm needlepoint is angled at 90 degrees as it emerges from the beak (Figure 2).

The instrument's beak is oriented in a buccolingual fashion and locked into place to measure that (Indian Patency Right Application Number, 201941043211) [18].

Probing Procedure

A mouth mirror was used for withdrawal and better visibility to avoid gingival puncturing in the buccal and palatal minor region of the gingiva, and the bills of the CAITN test were inserted into the deepest part of the cervically scraped spot. Some portion of the tooth is opposite to the long hub of the tooth to record buccolingual measurements [19,20]. The depth of the cervical abrasion may be calculated by comparing the reading on the CAITN probe scale with the mesiodistal and buccolingual measurements of the affected tooth, all of which are standardized.

Development of the CAITN index

The CAITN index was created using data from prior extensive research [16-20] to inform treatment choices. The authors examined 840 teeth from 30 participants, with a mean age of 48.4 years, and with or without cervical abrasions [16,19]. Using a CAITN probe [18], we recorded the buccolingual dimension of each tooth. Sensitivity was measured by patients' reactions to an air blast that was administered for one second to each tooth at a distance of 1 cm. The data acquired by the investigator was compared to the information provided by an endodontist who specializes in treating cervical abrasions. Also, the optimal cutoff value of the buccolingual measurement was obtained, which was then utilized to forecast the different types of TN, by the use of a receiver operating characteristics curve. The area under the curve (AUC) measures how well a model can predict TN from buccolingual data, and a higher AUC indicates a more accurate prediction of TN [20].

CAITN Index Rules

The index tooth is to be excluded when they are indicated for extraction for any cause and/or with carious lesions in the cervical region. Developmental malformations such as amelogenesis imperfect, enamel hypoplasia, and dens invaginations were also excluded.

1. To qualify for scoring, two or more healthy teeth must be present in any one of the three areas.

2. If just one of the two index teeth are present or included in an area, then that region's score is based on the evaluation of the remaining index tooth.

3. If both of the index teeth are missing or cannot be counted, the greatest possible score is taken from the remaining teeth in that area.

4. Whether or not any teeth remain in a given area determines the score given to that area; a single nonfunctional tooth is also considered to be absent from the area.

5. If there is just one tooth in that area, it is treated the same as if it were in the next area and must follow its regulations.

Criteria for scoring and classification of TN

The CAITN scores and their corresponding criteria for scoring are tabulated in Table 1.

**Table 1:** Criteria for scoring CA. CA, cervical abrasion

Tooth	CA score	Description	Buccolingual dimension (mm)
CA score: maxillary arch			
Molars	CA0	No CA/improper brushing technique	11-10
	CA1	Mild CA	9.9-9.5
	CA2	Moderate CA	9.4-6.5
	CA3	Moderately severe CA	6.5-3.5
	CA4	Severe CA	<3.5
	CAX	Excluded	-
Premolars	CA0	No CA/improper brushing technique	9-8
	CA1	Mild CA	7.9-7.5
	CA2	Moderate CA	7.4-5.5
	CA3	Moderately severe CA	5.4-3.5
	CA4	Severe CA	<3.5
	CAX	Excluded	-
Canine	CA0	No CA/improper brushing technique	8-7
	CA1	Mild CA	6.9-6.5
	CA2	Moderate CA	6.4-5.5
	CA3	Moderately severe CA	5.4-3.5
	CA4	Severe CA	<3.5
	CAX	Excluded	-
Incisors	CA0	No CA/improper brushing technique	7-6
	CA1	Mild CA	5.9-5.5
	CA2	Moderate CA	5.4-3.5
	CA3	Moderately severe CA	3.4-2.5
	CA4	Severe CA	<2.5
	CAX	Excluded	-
CA score: mandibular arch			
Molars	CA0	No CA/improper brushing technique	9-8
	CA1	Mild CA	7.9-7.5
	CA2	Moderate CA	7.4-4.5
	CA3	Moderately severe CA	4.4-2.5
	CA4	Severe CA	<2.5
	CAX	Excluded	-
Premolars	CA0	No CA/improper brushing technique	8-7
	CA1	Mild CA	6.9-6.5
	CA2	Moderate CA	6.4-4.5
	CA3	Moderately severe CA	4.4-2.5
	CA4	Severe CA	<2.5
	CAX	Excluded	-
Canine	CA0	No CA/improper brushing technique	8-7
	CA1	Mild CA	6.9-6.5
	CA2	Moderate CA	6.4-5.5
	CA3	Moderately severe CA	5.5-3.5
	CA4	Severe CA	<3.5
	CAX	Excluded	-
Incisors	CA0	No CA/improper brushing technique	6-5
	CA1	Mild CA	4.9-4.5
	CA2	Moderate CA	4.4-3.5
	CA3	Moderately severe CA	3.4-2.5
	CA4	Severe CA	<2.5

Based on the CAITN score, the recommended TN were classified, as shown in Table 2.

**Table 2:** Criteria for scoring CAITN. TN, treatment needs; CA, cervical abrasion

CA TN score	Description	CA score	Level of prevention	Mode of intervention
TN0	No TN/proper tooth brushing instructions TN	0	Primordial or primary	Health promotion
TN1	Minimal TN (desensitizing agents application)	1	Primary	Specific protection
TN2	Basic restorative TN (composite/glass ionomer cement application)	2	Primary	Specific protection
TN3	Advanced restorative TN (root canal therapy + composite/crown application)	3	Secondary	Early diagnosis and prompt treatment
TN4	Advanced Restorative TN (Root Canal Therapy + Composite/Crown Application) or Rehabilitative TN (Extraction + Removable/Fixed Prosthesis)	4	Secondary or Tertiary	Early diagnosis and prompt treatment or disability limitation and rehabilitation
TNX	Excluded TN	X	Excluded	Excluded

Limitations

There are various misconceptions concerning the etiology and progression of cervical abrasion, and there are significant variances in how dentists diagnose and treat cervical lesions. The findings of the study could be more susceptible to conflicting results due to a smaller sample size and individual variations in the buccolingual measurements of the tooth. Rather, noncarious cervical lesions may be caused by a combination of variables related to tooth brushing and masticatory forces. Most of these lesions are symptomatic, prompting patients to complain about their teeth being sensitive. Because the study was carried out in those patients attending specialty care clinic, the findings cannot be applied to the general population. Also, the data obtained from the patients such as their diet, brushing habits, and other oral hygiene practices would contribute to errors of accuracy.

## Conclusions

Within the constraints of the review, CAITN appears to be the most practicable method for routinely screening and recording cervical abrasion lesions that were previously unavailable to practitioners. For healthcare planning, the data serves as the foundation for determining general population TN. In clinical practice, the process provides a screening method for establishing the appropriate amount of intervention as well as a tool for monitoring long-term illness. The index provides epidemiologists, public health professionals, and practitioners with a practical technique for determining the TN of cervical abrasions when used appropriately. These multifactorial lesions, which present in a wide range of clinical manifestations, require a well-balanced and detailed therapeutic strategy. A reasonably standardized, yet reliable restorative method would make their clinical care easier. Furthermore, when explaining lesions to their staff and patients, a simplified clinical classification might be helpful for general dentists.
